# Supplementary material for: Content-rich biological network constructed by mining PubMed abstracts
Source: BMC Bioinformatics. 2004 Oct 8;5:147. doi: 10.1186/1471-2105-5-147 (PMC528731; doi:10.1186/1471-2105-5-147)
Supplement: Additional File 2 — The original results of the above study (non-essential files are deleted to keep the file size under the limit set by BMC bioinformatics). [file 1471-2105-5-147-S2.bz2 › chilibotAdditionalFile2/dip05/730ID11983079E18088/html/HSCB_NIFU.html]

 


 **HSCB** and **NIFU** 
  
Found 2 abstracts in PubMed, retrieved 2.  
 

 What does Google say? 
 PDF only 
| .edu only 

---

**Interactive relationship** (e.g. stimulation, inhibition, etc)

**Non-interactive relationship** (e.g. studied together, co-existance, homology, etc.)

- A charge mutant containing the motif sequence of  **NifU** , IscU V102E, did not bind the ATP or ADP states of Hsc66 but did bind Hsc20  [ **HSCB** ]  and weakly stimulated Hsc66 ATPase in the presence of the co chaperone.  Ref: 12871959 J Biol ChemJ Biol Chem,
